# Relationship between post-COVID-19 symptoms and daily physical activity

**DOI:** 10.3389/fresc.2025.1646093

**Published:** 2025-09-15

**Authors:** Antonio Sarmento, Sandra Webber, Shelley Sargent, Brenda Tittlemier, Diana C. Sanchez-Ramirez

**Affiliations:** ^1^ Department of Respiratory Therapy, University of Manitoba, Winnipeg, MB, Canada; ^2^ Department of Physical Therapy, University of Manitoba, Winnipeg, MB, Canada; ^3^ Rehabilitation Physiotherapy Department, Health Sciences Centre, Winnipeg, MB, Canada

**Keywords:** post COVID “long-haulers”, post COVID syndrome, post COVID symptoms, physical activity, wearables

## Abstract

**Background:**

Exertion-intolerant symptoms common in post-COVID-19 syndrome (PCS), often resembling myalgic encephalomyelitis/chronic fatigue syndrome (ME/CFS), challenge conventional rehabilitation and highlight the need for research into the poorly understood relationship between PCS symptoms and physical activity.

**Objectives:**

We aimed to investigate the longitudinal associations between PCS symptoms and physical activity (same and following day), while accounting for the presence of myalgic encephalomyelitis/chronic fatigue syndrome (ME/CFS) symptoms. Additionally, to compare the characteristics and outcomes of PCS patients with and without ME/CFS symptoms.

**Methods:**

Adults with PCS participated in an in-person evaluation that included assessment of dyspnea (Borg scale), fatigue (Fatigue Severity Scale), ME/CFS symptoms screening (DePaul Symptom Questionnaire), and functional capacity. Participants were also instructed to complete a daily PCS symptoms survey and wear a smartwatch for a week to track daily physical activity (step count).

**Results:**

Eighteen individuals with PCS (78% females, 51 ± 11 years) participated in the study, averaging 4,067 steps per day (95%CI 3,638–4,497) over 117 days of valid data. Individuals with ME/CFS symptoms (*n* = 11) reported more severe PCS symptoms and had lower functional capacity than those without ME/CFS symptoms. After adjusting for ME/CFS symptoms, greater dizziness was associated with fewer steps on the same [OR 0.94 (95%CI 0.88–0.99), *p* = 0.026] and following day [OR 0.91 (95%CI 0.84–0.98), *p* = 0.016]. Lower levels of fatigue [OR 0.69 (95%CI 0.49–0.99), *p* = 0.043] and chest pain [OR 0.76 (95%CI 0.57–0.99), *p* = 0.048] were associated with walking ≥5,000 steps on the previous day.

**Conclusion:**

Regardless of the presence of ME/CFS symptoms, dizziness was negatively associated with physical activity on both the same and following day in PCS individuals. Additionally, lower levels of fatigue and chest pain were linked to walking 5,000 steps or more the previous day.

**Impact:**

These results provide insights into the relationships between symptoms and daily physical activity in PCS, which can help tailor interventions and improve the management of this condition. This research also highlights the value of using wearable devices and smartphone apps to collect data for monitoring individuals with PCS over time.

## Introduction

The long-term impact of SARS-CoV-2 infection, known as post-COVID-19 syndrome (PCS), is characterized by a range of symptoms that continue for 12 weeks or more after the acute phase of illness (1). This syndrome imposes a substantial burden on affected individuals, making everyday activities more challenging and impairing their functional capacity and quality of life (2–4).

PCS symptoms may be episodic in nature, causing health challenges that fluctuate or relapse over time, leading participants to experience good and bad days in terms of their illness and limitations (5). Literature suggests that a complex interplay of multiple factors may cause PCS and its wide range of symptoms (6–8), which can impact physical activity and lead to physical deconditioning (4, 9, 10). Although the exact mechanisms are still under investigation, persistent inflammation, immune dysregulation, endothelial dysfunction, and tissue damage have been identified as key contributing factors (7, 11, 12) Dysregulation of the cardiovascular and autonomic systems may play a role in the development of PCS symptoms, particularly contributing to shortness of breath, fatigue, chest pain, and dizziness (13, 14). Vestibular dysfunction resulting from COVID-19 may also cause dizziness, balance impairments, and anxiety about potential falls, all of which can limit daily activities (15–17). Additionally, studies have demonstrated that many individuals with PCS exhibit symptoms consistent with myalgic encephalomyelitis/chronic fatigue syndrome (ME/CFS), including severe fatigue, post-exertional malaise, and cognitive dysfunction (18, 19). These ME/CFS-related symptoms, which are often exacerbated by physical or mental exertion, contribute to reduced exercise tolerance and make conventional rehabilitation approaches unsuitable (20).

There is a critical need to study the relationship between PCS symptoms and physical activity, as it remains insufficiently understood. In this context, continuous health monitoring can effectively help achieve this purpose (21). The recent integration of commercial wearable devices and their associated smartphone apps into daily life offers a valuable tool to track and adjust activity levels, helping individuals manage and identify PCS based on their own data. Although wearable fitness trackers (e.g., smartwatches) have been increasingly used to monitor step counts, sedentary behavior, and cardiovascular parameters before and during the COVID-19 pandemic (22–24), literature still lacks longitudinal studies assessing how PCS symptoms affect the daily physical activity of these individuals. Therefore, this study aimed to investigate the longitudinal associations between PCS symptoms and physical activity (same and following day), while accounting for the presence of ME/CFS symptoms. Additionally, it compared the characteristics and outcomes of PCS patients with and without ME/CFS symptoms.

## Methods

### Design and participants

This prospective short-term longitudinal exploratory study was approved by the research ethics committee of the University of Manitoba [HS25555(B2022:056)] and conducted according to the Declaration of Helsinki. All participants included in this research signed the informed consent form.

A convenience sample was recruited through public advertising and social media. Inclusion criteria were age ≥18 years, persistence of symptoms ≥3 months after confirmed or suspected COVID-19 infection (25), and access to a smart device (phone or tablet) and internet service. Those who reported full recovery of symptoms, limited ability to walk independently, acute lung or heart conditions, hearing or visual impairments, and those who could not complete basic tasks on the smart device (e.g., searching, opening, and closing an app) were excluded.

### Assessments and data collection

Individuals were screened for eligibility via telephone. The study, conducted between Nov 2022 and April 2023, involved two components: in-person assessment (RespirabilityLab) and home-based remote monitoring.

#### In-person assessment

Anthropometric data (height, weight, and body mass index) were initially collected during the in-person assessment, followed by a validated and reliable assessment of dyspnea using the modified Borg scale (0–10 points, where 10 indicating maximal perceived exertion) (26) and fatigue, using the Fatigue Severity Scale (27). The latter is a self-reported instrument that assesses fatigue severity regarding daily activities using a 7-point Likert scale; total scores are determined as the average of responses and range between 1 (no impact) and 7 (greater impact of fatigue symptoms). The visual analog scale of the Fatigue Severity Scale was also used to evaluate overall fatigue (0 indicated “very alert” and 10 “extremely fatigued”) (28). The FSS is a valid, reliable, and responsive instrument for assessing fatigue severity (Cronbach's α > 0.90; ICC 0.80–0.90) (29, 30).

Participants were also screened for ME/CFS symptoms using the DePaul Symptom Questionnaire short-form (DSQ-SF) (31) by rating the frequency and severity of 14 symptoms on a 5-point Likert scale. The DSQ short-form demonstrates strong psychometric properties, it can accurately classify individuals with Long COVID and ME/CFS with high degrees of sensitivity and specificity (32). To fulfill the Canadian ME/CFS criteria (33), which is the most complete case definition, the participant should score at least a 2/5 frequency and 2/5 severity threshold on the DSQ-SF domains related to fatigue, post-exertional malaise, unrefreshing sleep, and neurocognitive impairment, and should list one symptom from at least two of the following domains: pain (either muscle pain or bloating), autonomic (either irritable bowel problems or feeling unsteady on your feet, like you might fall), neuroendocrine (either sensation of cold limbs or feeling hot or cold for no reason), and immune (either flu-like symptoms or some smells, foods, medications, or chemicals make you feel sick). The total score for each DSQ-SF domain was also averaged and multiplied by 25 to create a 100-point composite score; values close to 100 represent more burden of symptoms (31, 34).

Two field tests were applied to assess functional capacity. Firstly, participants performed the 1-minute sit-to-stand test (1MSTST) using a chair without armrests. The 1MSTS demonstrates good reliability, validity, and responsiveness for evaluating functional exercise capacity in clinical populations, with strong test-retest agreement and moderate correlations to other recognized functional assessments (35, 36). They were asked to fold their arms across their chest and stand up and sit down as quickly as possible for one minute. The best performance out of the three attempts was included in the analyses. Absolute and percentage of predicted values of the trial with the greatest number of repetitions were included for analysis (37). Secondly, the six-minute walk test (6MWT) was conducted by asking participants to walk as far as possible without running in a 30-meter corridor during six minutes (38). The distance covered was recorded in meters and presented as absolute and percentage of predicted values (37, 38). The 6MWT demonstrates moderate to excellent psychometric properties including validity, reliability, and responsiveness to change which make it clinically useful and applicable for assessing functional capacity in a variety of populations (39). Lastly, lung function (forced vital capacity, forced expiratory volume in the first second and peak expiratory flow) was assessed using a SpiroBank Smart spirometer (MIR, Rome, Italy) according to ATS/ERS recommendations (40). Data were compared to reference values for the Canadian population (41).

#### Remote monitoring

A Garmin Venu Sq® smartwatch (Garmin, Kansas, USA), provided during the in-person assessment, was paired via Bluethooth with the participant's smartphone and connected to the Garmin Connect and Labfront Companion (Labfront, Boston, USA) applications. The feasibility of this wearable device to acquire data throughout the day was validated in a previous study with individuals with PCS (42). After instructions about daily data synchronization, participants were asked to wear the smartwatch on the non-dominant wrist for seven days during waking hours while continuing with their regular activities. Participants completed an electronic questionnaire (using the Labfront Companion application) daily, reporting the severity (0 “none” to 10 “very severe”) of some of the most prevalent PCS symptoms: shortness of breath at rest and during activities, chest pain, dizziness, and fatigue (43). Participants were able to remove the watch overnight for charging and were instructed to avoid using the smartwatch during showering or swimming.

For data to be considered valid, participants should have worn the smartwatch for at least eight consecutive waking hours. The following variables were calculated: total wear time (in hours) during the day and during waking hours (i.e., between 6am and 10pm), daily step count, and HR (average, minimum, and maximum). Daily step count was used to determine physical activity levels (44) and was presented using two variables: steps acquired the same day and steps acquired the following day.

### Statistical analysis

Demographic data, lung function, and DSQ-SF scores were compared between individuals with and without ME/CFS symptoms using the Mann–Whitney test. Valid data on wear time, daily step count, heart rate (HR), and symptoms, clustered at the participant level, were compared between individuals with and without ME/CFS symptoms. Generalized Estimating Equations (GEEs) were applied to examine the associations between PCS symptoms and daily step count (same and following day), using three models with data clustered at the participant level and adjusted for sex and age: Model 1 assessed associations for each symptom individually, Model 2 considered all symptoms simultaneously, and Model 3 built on Model 2 by further adjusting for the presence of ME/CFS symptoms. Furthermore, GEEs were applied to examine the associations between walking ≥5,000 steps/day (44) (yes/no) and symptom severity the next day. Three models were developed, each building upon the previous one: Model 1 adjusted for same day symptom, Model 2 adjusted for sex and age, and Model 3 further adjusted for the presence of ME/CFS symptoms (Model 3).

Data were analyzed using SPSS software (IBM Corp., NY, USA) and presented as means ± standard deviations, estimated marginal means (95% confidence intervals), and odds ratio (OR). Significance was set at *p* < 0.05.

## Results

Eighteen individuals with PCS (78% females, 51.16 ± 11.03 years, and 29.83 kg/m^2^) were included in the study (Table 1). Six participants worked in office-based roles (graphic designer, supervisor, actuary, library technician, sales, accountant), 4 were healthcare providers (2 nurses, 1 paramedic, 1 massage therapist), 3 were teachers, 2 were retired, 1 was an engineer, 1 worked in aerospace manufacturing, and occupational information was missing for one participant. Twelve participants reported having one or more pre-existing comorbidities, the most common being asthma, hypothyroidism, diabetes, migraine, obstructive sleep apnea, hypertension arterial, and orthostatic hypotension. Fifteen individuals wore the smartwatch for 7 days, while the other three individuals wore it for 6, 4, and 2 days, totaling 117 days of valid data. Overall, participants wore the watch for a mean time of 18.83 hours per day and walked a mean of 4,067 steps daily (95%CI 3,638–4,497). The most severe symptoms reported were shortness of breath during activity and fatigue (Table 2). Dizziness was reported by 78% (*n* = 14) of the participants.

**Table 1:** Characteristics of study participants.

Variables	All (*n* = 18)	ME/CFS symptoms		
		Yes (*n* = 11)	No (*n* = 7)	*p*-value
Sex *n* (%)				
Male	4 (22)	1 (9)	3 (43)	<0.001
Female	14 (78)	10 (91)	4 (57)	
Age (years)	51.16 ± 11.03	48.18 ± 11.30	55.85 ± 8.86	0.211
Body mass index (kg/m^2^)	29.83 ± 6.38	31.57 ± 7.19	27.10 ± 3.84	0.179
Lung function (%pred)				
FVC	103.45 ± 21.47	100.51 ± 26.18	107.65 ± 12.88	0.270
FEV_1_	98.46 ± 20.20	94.12 ± 22.17	105.28 ± 15.77	0.104
Peak expiratory flow	97.94 ± 28.14	87.22 ± 25.60	114.78 ± 24.71	0.035
Borg scale	2.19 ± 1.34	2.68 ± 1.19	1.43 ± 1.27	0.069
Fatigue severy scale				
Total score	5.51 ± 1.03	5.90 ± 0.56	4.90 ± 1.34	0.096
Visual analog scale	4.53 ± 2.87	5.18 ± 3.25	3.51 ± 1.94	0.237
DePaul symptom questionnaire				
Fatigue/extreme tiredness	77.08 ± 18.81	85.23 ± 16.60	46.00 ± 31.95	0.020
Next day soreness or fatigue	68.75 ± 30.09	78.41 ± 20.98	53.57 ± 37.30	0.151
Tiredness after minimum exercise	63.19 ± 26.59	69.32 ± 23.96	53.57 ± 37.30	0.328
Feeling unrefreshed in the morning	77.78 ± 21.25	81.82 ± 18.00	71.43 ± 25.73	0.425
Muscle pain or aching	65.97 ± 25.29	78.41 ± 15.90	46.43 ± 25.73	0.015
Bloating	70.83 ± 20.11	78.41 ± 15.90	58.93 ± 21.30	0.069
Problems remembering things	53.47 ± 20.02	61.36 ± 18.92	41.07 ± 15.67	0.056
Difficulty paying attention	43.75 ± 33.28	48.86 ± 31.35	35.71 ± 37.10	0.479
Irritable bowel problems	44.44 ± 35.41	51.14 ± 34.21	33.93 ± 37.30	0.375
Feeling unsteady like you might fall	45.83 ± 26.43	54.55 ± 28.10	32.14 ± 17.47	0.069
Cold limbs	42.36 ± 31.25	57.95 ± 27.54	17.86 ± 18.90	0.006
Feeling hot or cold for no reason	50.00 ± 27.79	57.95 ± 29.19	37.50 ± 21.65	0.126
Flu-like symptoms	36.81 ± 26.59	46.59 ± 28.00	21.43 ± 15.67	0.056
Smells, foods, medications, or chemicals make you feel sick	38.19 ± 33.89	47.43 ± 31.53	23.21 ± 34.18	0.151
1-minute sit-to-stand test				
Number of repetitions	18.89 ± 6.70	16.27 ± 6.62	23.00 ± 4.69	0.033
Percentage of predicted value^a^	48.71 ± 17.04	41.34 ± 17.17	60.28 ± 8.75	0.016
6-minute walk test				
Distance (meters)	333.74 ± 110.28	267.90 ± 84.61	437.19 ± 45.81	<0.001
Percentage of predicted value	60.39 ± 20.11	49.96 ± 16.85	76.76 ± 12.66	0.002

Data shown as mean ± standard deviation or absolute and relative frequencies. ME/CFS, myalgic encephalomyelitis/chronic fatigue syndrome; FVC, forced vital capacity; FEV_1_, forced expiratory volume in the first second; %pred, percentage of predicted.

^a^ Percentile 50 (37).

**Table 2:** Smartwatch wear time, physical activity (daily step count), heart rate, and PCS symptoms of study participants.

Variables	All (*n* = 18)	ME/CFS symptoms		
		Yes (*n* = 11)	No (*n* = 7)	*p*-value
Watch wear time (hours)				
All day	17.83 (16.82–18.84)	17.22 (15.18–19.52)	18.70 (16.02–21.83)	0.416
Between 6am and 10pm	13.46 (13.00–13.99)	12.93 (11.98–13.96)	14.27 (13.39–15.21)	0.051
Step count (number)				
Daily average	4,067 (3,638–4,497)	3,683 (2,766–4,497)	4,644 (3,591–6,007)	0.237
Heart rate (bpm)				
Minimum	59.30 (58.00–60.61)	60.69 (57.07–64.54)	57.24 (53.95–60.74)	0.180
Average	81.49 (79.98–82.99)	83.31 (79.13–87.72)	78.46 (74.79–82.32)	0.095
Maximum	122.49 (120.55–124.44)	122.70 (118.71–126.82)	121.82 (116.75–127.11)	0.795
Symptoms_SD_ (0–10^a^)				
SOB at rest	2.05 (1.69–2.42)	2.52 (1.44–3.60)	1.28 (0.60–1.96)	0.056
SOB during activity	3.31 (2.84–3.78)	4.15 (2.79–5.51)	1.84 (0.91–2.76)	0.006
Chest pain	1.64 (1.31–1.97)	2.13 (1.14–3.12)	0.63 (0.13–1.14)	0.008
Dizziness	1.88 (1.48–2.29)	2.69 (1.60–3.77)	0.72 (0.04–1.49)	0.004
Fatigue	4.15 (3.63–4.68)	5.35 (3.76–6.94)	2.46 (1.73–3.19)	0.001

Data shown as estimated marginal mean (95% confidence interval). SD, same day; SOB, shortness of breath; bpm, beats per minute; ME/CFS, myalgic encephalomyelitis/chronic fatigue syndrome.

^a^ A higher number indicated greater severity.

Individuals with ME/CFS symptoms (*n* = 11) performed worse on the 6MWT (*p* < 0.001) and 1MSTST (*p* = 0.033) than those without ME/CFS symptoms. They also reported significantly more shortness of breath during activity (*p* = 0.006), chest pain (*p* = 0.008), dizziness (*p* = 0.004), and fatigue (*p* = 0.001) (Table 2).

Increased severity of all symptoms was independently associated with a lower step count on the same day (all *p* < 0.05). However, when including all symptoms in the same model, only dizziness remained significantly associated even after adjusting for ME/CFS symptoms [OR 0.94 (95%CI 0.88–0.99), *p* = 0.026]. Greater dizziness was also significantly associated with lower step count on the following day in individuals with PCS, independent of ME/CFS symptoms [OR 0.91 (95%CI 0.84–0.98), *p* = 0.016] (Table 3). Moreover, lower levels of fatigue [OR 0.69 (95% CI 0.49–0.99), *p* = 0.043] and chest pain on the following day [OR 0.76 (95%CI 0.57–0.99), *p* = 0.048] were associated with walking ≥5,000 steps/day independent of the magnitude of these symptoms on the previous day, age, sex, and presence of ME/CFS symptoms (Table 4).

**Table 3:** Associations between PCS symptoms and physical activity (daily step count) in the same and following days adjusted for sex, age and presence of ME/CFS symptoms.

Variables	Model 1 (crude)	Model 2	Model 3
	OR (95%CI)	OR (95%CI)	OR (95%CI)
Steps_SD_			
SOB at rest_SD_	**0.90 (0.83**–**0.98)**	0.97 (0.89–1.06)	0.98 (0.89–1.07)
SOB during activity_SD_	**0.91 (0.84**–**0.98)**	0.96 (0.88–1.05)	0.95 (0.86–1.05)
Chest pain_SD_	**0.90 (0.81**–**0.99)**	0.95 (0.87–1.04)	0.94 (0.87–1.03)
Dizziness_SD_	**0.90 (0.84**–**0.98)**	**0.94 (0.89**–**0.99)**	**0.94 (0.88**–**0.99)**
Fatigue_SD_	**0.95 (0.89**–**0.99)**	1.01 (1.01–1.03)	1.01 (0.94–1.07)
Steps_ND_			
SOB at rest_SD_	0.95 (0.86–1.04)	1.03 (0.95–1.11)	1.03 (0.95–1.12)
SOB during activity_SD_	0.93 (0.85–1.02)	0.95 (0.87–1.04)	0.94 (0.85–1.05)
Chest pain_SD_	**0.87 (0.78**–**0.98)**	0.89 (0.80–1.01)	0.89 (0.80–1.01)
Dizziness_SD_	**0.90 (0.83**–**0.98)**	**0.91 (0.84**–**0.98)**	**0.91 (0.84**–**0.98)**
Fatigue_SD_	0.99 (0.93–1.05)	1.04 (0.97–1.10)	1.04 (0.97–1.11)

SOB, shortness of breath; SD, same day; ND, next day; OR, odds ratio; CI, confidence interval; ME/CFS, myalgic encephalomyelitis/chronic fatigue syndrome; generalized estimating equations (clustered at the patient level) with number of steps SD and ND as dependent variables, adjusted for sex and age, using three models: Model 1 (crude): each symptom was analyzed individually; Model 2: included all symptoms in a single model, and Model 3 built on Model 2 by further adjusting for ME/CFS symptoms (yes/no). Statistically significant results are in bold (*p* < 0.05).

**Table 4:** Associations between physical activity (daily step count) and PCS symptoms in the following day adjusted for age, sex, same day symptom severity, and presence of ME/CFS symptoms.

Variables	Model 1 (crude)	Model 2	Model 3
	OR (95% CI)	OR (95% CI)	OR (95% CI)
SOB at rest_ND_			
Step count_SD_ (≥ 5,000)^a^	0.69 (0.33–1.46)	0.70 (0.33–1.48)	0.70 (0.35–1.39
SOBRest_SD_	1.09 (0.86–1.38)	1.23 (0.87–1.72)	1.211 (0.83–1.76)
Age	–	0.98 (0.95–1.01)	0.99 (0.97–1.03)
Sex (female)^a^	–	0.39 (0.09–1.75)	0.33 (0.07–0.82)
ME/CFS symptoms (yes)^a^	–	–	3.86 (1.61–9.29)
SOB during activity_ND_			
Step count_SD_ (≥ 5,000)^a^	0.93 (0.71–1.22)	0.96 (0.80–1.15)	0.96 (0.80–1.14)
SOB activity_SD_	2.69 (2.60–2.77)	2.70 (2.63–2.77)	2.61 (2.50–2.72)
Age	–	0.99 (0.99–0.99)	0.99 (0.99–1.00)
Sex (female)^a^	–	0.97 (0.80–1.18)	0.90 (0.72–1.13)
ME/CFS symptoms (yes)^a^	–	–	1.24 (1.04–1.46)
Chest pain_ND_			
Step count_SD_ (≥ 5,000)^a^	0.73 (0.58–0.91)	0.76 (0.57–1.02)	**0.76 (0.57**–**0.99)**
Chest pain_SD_	2.27 (2.02–2.54)	2.27 (1.98–2.59)	2.17 (1.84–2.57)
Age	–	1.00 (0.99–1.01)	1.01 (0.99–1.01)
Sex (female)^a^	–	0.91 (0.63–1.30)	0.87 (0.59–1.30)
ME/CFS symptoms (yes)^a^	–	–	1.26 (1.02–1.57)
Dizziness_ND_			
Step count_SD_ (≥ 5,000)^a^	1.04 (0.73–1.48)	0.58 (0.39–0.85)	1.25 (0.58–2.71)
Dizziness_SD_	2.56 (2.30–2.86)	2.57 (2.27–2.91)	1.43 (1.01–2.02)
Age	–	1.01 (1.01–1.02)	0.99 (0.96–1.03)
Sex (female)^a^	–	2.14 (1.59–2.87)	2.32 (1.09–5.00)
ME/CFS symptoms (yes)^a^	–	–	3.25 (1.42–7.40)
Fatigue_ND_			
Step count_SD_ (≥ 5,000)^a^	0.81 (0.47––1.41)	0.69 (0.46–1.02)	**0.69 (0.49**–**0.99)**
Fatigue_SD_	2.43 (2.21–2.68)	2.44 (2.27–2.61)	2.50 (2.39–2.76)
Age	–	0.99 (0.98–1.01)	0.99 (0.98–1.01)
Sex (female)^a^	–	1.42 (1.07–1.87)	1.51 (1.16–1.97)
ME/CFS symptoms (yes)^a^	–	–	0.74 (0.56–0.99)

SOB, shortness of breath; SD, same day; ND, next day; OR, odds ratio; CI, confidence interval; min, minutes; ME/CFS, myalgic encephalomyelitis/chronic fatigue syndrome.

^a^ <5,000 steps per day (i.e., sedentary behavior), absence of ME/CFS symptoms, and male sex were considered the categories of reference. Generalized estimating equations (clustered at the participant level) with symptom severity ND (0–10) as dependent variable. Model 1 (crude): included step count SD ≥ 5,000 (yes/no) adjusted for SD symptom; Model 2: Model1 adjusted for sex and age; Model 3: Model 2 adjusted for ME/CFS symptoms (yes/no). Statistically significant results are in bold.

## Discussion

To the best of our knowledge, this is the first study that has investigated the longitudinal associations between PCS symptoms and physical activity in individuals with PCS using wearable devices, accounting for the presence of ME/CFS symptoms. Our results indicated that PCS individuals with ME/CFS symptoms presented greater SOB during activity, chest pain, dizziness, and fatigue, along with lower functional capacity compared to those without ME/CFS symptoms. Although the exact causes remain unclear, post-infectious fatigue has been linked to several factors, including chronic immune activation, autoimmunity, and mitochondrial dysfunction. However, the presence of ME/CFS symptoms did not affect the study findings, which revealed that dizziness was negatively associated with daily step count on the same and following day. Additionally, walking ≥5,000 steps was linked to lower levels of fatigue and chest pain the following day.

Research has found that dizziness is highly prevalent in PCS (45, 46). Evidence suggests that individuals experiencing dizziness often make significant adjustments to their daily routines, encounter physical performance limitations, and may reduce or avoid physical activities due to fear-avoidance behavious (47, 48). However, the association of dizziness with physical function and physical activity have been minimally explored in PCS, with studies relying mainly on questionnaires or self-reports (45, 49). A recent multicenter study (49) demonstrated that dizziness in individuals with PCS was linked to higher degrees of dependency, limitations in functionality and challenges in activities of daily living (e.g., self-care, home management, recreation, and travel). Perceived disability due to dizziness and fear of falling was also reported in young adults with PCS (50). In this study, we found a correlation between increased dizziness and reduced step count on the same and following day. This finding aligns with results from research on adolescent athletes post-concussion, who were monitored using smartwatches. In that study (51), self-reported dizziness at the initial assessment was associated with lower daily step count (mean reduction of 1,035 steps per day) in the following two weeks, suggesting that dizziness may contribute to sustained low levels of physical activity.

The influence of physical activity on the emergency of PCS symptoms has been addressed in recent studies (9, 52, 53). A large retrospective cohort (52) reported that being physically active during the pandemic was associated with a lower prevalence of symptoms of PCS. Similarly, other retrospective cohorts observed that sedentary behavior was associated with a 25% higher risk of developing at least one symptom of PCS (53), while high physical activity over the year prior to a COVID-19 infection was a protective factor against experiencing fatigue and chest pain (54). Despite the importance of these results, physical activity was self-reported and subjected to memory bias, which could overestimate vigorous activity and underestimate sedentary time (55). Furthermore, it remains unclear whether the real-time reciprocal relationships between physical activity and symptoms persist in individuals with established PCS, largely because inactivity in a day-to-day basis might contribute to and/or result from PCS (10, 56). PCS symptoms (43) can impact functional capacity and physical activity levels (18, 57), potentially explaining the decreased performance in the 6MWT and 1MSTST and the lower activity levels identified in our study (18, 58, 59) Additionally, reduced physical activity may lead to physical deconditioning (4, 9, 10), reduced aerobic capacity and muscle strength (60), further diminishing functional capacity in this population (52, 61). Conversely, physical activity may exacerbate symptoms such as severe fatigue, post-exertional malaise, and cognitive dysfunction in PCS individuals with ME/CFS symptoms (18, 19). This may result in reduced exercise tolerance, making conventional rehabilitation approaches unsuitable (20).

In this context, the associations between walking at least 5,000 steps/day and lower levels of fatigue and chest pain on the following day (regardless of ME/CFS symptoms), may provide valuable insights into the potential cycle of symptom exacerbation and physical deconditioning in individuals with PCS. Lippi et al. (10) suggested that individuals with PCS are more likely to reduce their physical activity levels after recovering from an acute infection; thus, increasing the risk of generating a continuous loop of symptom worsening. Individuals with PCS spend a shorter time walking up to six months after an acute infection (62), whereas those who are more physically active present with less fatigue, better performance on the 6MWT, higher number of repetitions in the 1MSTST, and higher handgrip muscle strength (63). Higher time in sedentary behavior and prolonged sitting without intermittent activity, as observed in COVID-19 survivors (64), may also potentially contribute to cardiovascular issues that could manifest as chest pain (65). Although different from sedentary behavior, inactivity may cause physical deconditioning, lowering the pain threshold and potentially increasing the likelihood of experiencing symptoms, such as non-cardiac chest pain (66).

The bidirectional relationships between the intensity of symptoms and physical activity observed in this study also has important implications for PCS management. Although more in-depth research is needed to elucidate the cycle of symptom exacerbation and physical deconditioning in PCS, comprehensive rehabilitation strategies (15, 16) addressing dizziness during rehabilitation might mitigate the fear avoidance behavior (67) and increase daily step count in the same and following days. Studies have demonstrated that vestibular rehabilitation can improve dizziness, balance, and mobility (68), while lower dizziness severity is correlated with higher physical activity levels (69). Consequently, increasing mobility to a minimum of a low active physical activity level (5,000 steps) could influence the reduction of chest pain and fatigue symptoms in the following day, assisting with recovery. Being physically active provides several health benefits, including protection against inflammation (70) and autoimmunity disorders (71), which are potentially linked to the development of PCS. Studies have shown a protective effect of a physically active lifestyle on COVID-19-related outcomes (72). Similarly, a meta-analysis of 23 studies (1,579 individuals) suggested that physical exercise-based rehabilitation programs were potential effective therapeutic strategies against PCS, as they resulted in significant improvements in dyspnea, fatigue, exercise capacity, and quality of life (73). As PCS patients may experience difficulty return to the pre-infection levels of physical activity (62), it may be advisable to cautiously encourage a gradual increase in the level of physical activity (at least from sedentary to low active) on a case-by-case basis, ensuring progression is safe and tailored to the severity of symptoms (74, 75). This approach can be integrated into routine clinical practice to help alleviate the burden of PCS symptoms. However, patient-tailored multidisciplinary support, along with greater attention from health care providers for early detection and proper management of PCS symptoms, are also essential to improve outcomes in this population (76).

### Limitations and strengths

While this study provides valuable insights, the findings should be interpreted with caution, as certain limitations may restrict their generalizability. First, we acknowledge that including participants with both confirmed and suspected COVID-19 symptoms may introduce heterogeneity into the sample. This approach was used to capture a broader range of experiences during the evolving pandemic, recognizing that diagnostic confirmation was not always accessible. According to the WHO (Global Surveillance for COVID-19: Interim guidance, 20 March 2020), a confirmed case is one with laboratory confirmation of COVID-19 infection regardless of symptoms, while a suspected (presumptive) case meets clinical and/or epidemiological criteria without laboratory confirmation. Both definitions have been accepted in the literature to ensure timely data collection and broader case identification (77). Second, pre-existing comorbidities and/or medication use may have influenced the reported symptoms. A summary of the main reported comorbidities was provided; however, information on medication use was not available. Although controlling for these factors was not feasible due to the small sample size, we acknowledge their relevance for interpreting the findings. Third, the relatively small sample, composed mainly of females and individuals with a low level of physical activity, may impact the generalizability of the findings. However, literature indicates that these characteristics are common in individuals with PCS, suggesting that the sample may properly represent the population (10). Also, generalized estimating equations were employed to control the type I error and to maintain accuracy in the estimated marginal means. Fourth, the use of convenience sampling via social media may have introduced selection bias, limiting the representativeness of the sample. However, many studies have successfully utilized social media to identify participants and collect data, demonstrating its practical value in reaching diverse populations (77, 78). Fifth, information about lifestyle, which may have influenced the physical activity level of participants, was not gathered; however, we believe this does not affect the findings since step count was used to objectively measure activity levels in all participants and examine their relationship with symptom severity. Sixth, post-exertional malaise was not assessed in detail (79) and the self-reported information obtained in this study only allowed identification of ME/CFS symptoms, as ME/CFS diagnosis requires a full clinical assessment (80). Also, may have led to over- or under-reporting of symptoms, but in the absence of a valid tool, this questionnaire has been commonly used in other studies exploring this condition in PCS (73). Last, some participants experienced technical difficulties during the synchronization of Garmin data with the third-party app, resulting in data loss. The development or identification of a new application with similar data transfer capabilities may help overcome this challenge.

Nevertheless, this is the first study that longitudinally assessed daily step counts using wearable devices in PCS individuals with and without ME/CFS symptoms. In the context of PCS research, smartwatches and smartphone apps have emerged as invaluable personalized tools for remotely assessing, monitoring, and understanding the complex array of symptoms experienced by affected individuals (21, 81). Furthermore, the ability to track daily step counts helps quantify physical activity, adding to the comprehensive view of functional status and monitoring of recovery progress (75). As shown in our study, integrating these data points in the same and following days may help patients, clinicians and researchers understand the temporal relationships between PCS symptoms and daily physical activity; thus, enhancing the ability to tailor interventions and improve management strategies for those with PCS (76, 82, 83). Future research should explore the underlying mechanisms linking PCS, ME/CFS symptoms, and tachycardia, as well as investigate potential therapeutic strategies to address them. Additionally, future studies should aim for larger and more balanced sample sizes to increase statistical power, enhance the generalizability of findings, enable more detailed subgroup analyses, and adjustment for potentially relevant factors.

### Summary of recommendations

PCS management should include rehabilitation strategies addressing dizziness and supporting an individualized increase in physical activity to at least a low active level (≥5,000 steps/day), ensuring progression is safe and tailored to the severity of symptoms. Wearable devices and their companion apps can be used to monitor step counts and symptom fluctuations, enabling personalized care. Clinician guidance and patient education on the benefits and precautions of physical activity in PCS are essential to effectively support recovery and improve outcomes.

## Conclusion

Regardless of the presence of ME/CFS symptoms, dizziness was negatively associated with physical activity on both the same and following day in PCS individuals. Additionally, lower levels of fatigue and chest pain were linked to walking 5,000 steps or more on the previous day. These findings offer important insights into the complex interplay between symptoms and daily physical activity in PCS, which can inform the development of targeted interventions and enhance the management of the condition. This research also highlights the value of using wearable devices and smartphone apps to collect data for monitoring individuals with PCS over time.

## Data Availability

The datasets used in this article are not publicly available due to participant confidentiality agreements. However, they may be obtained from the corresponding author upon reasonable request and with the necessary ethical approvals. Requests to access the datasets should be directed to diana.sanchez-ramirez@umanitoba.ca.
